# Essential information for neurorecovery clinical trial design: trajectory of global disability in first 90 days post-stroke in patients discharged to acute rehabilitation facilities

**DOI:** 10.1186/s12883-023-03251-1

**Published:** 2023-06-20

**Authors:** Shayandokht Taleb, Jenny Ji-hyun Lee, Pamela Duncan, Steven C. Cramer, Mersedeh Bahr-Hosseini, Michael Su, Sidney Starkman, Gilda Avila, Arielle Hochberg, Scott Hamilton, Robin A. Conwit, Jeffrey L. Saver

**Affiliations:** 1 Department of Neurology, David Geffen School of Medicine, UCLA, Los Angeles, USA; 2 Department of Neurology, Kaiser Permanente Los Angeles Medical Center, Los Angeles, USA; 3 Department of Neurology, Wake Forest School of Medicine, Winston-Salem, USA; 4 Departments of Emergency Medicine and Neurology, David Geffen School of Medicine at UCLA, Los Angeles, USA; 5 BrainQ Technologies Ltd, Jerusalem, Israel; 6 Department of Neurology, Stanford University, Stanford, USA; 7 National Institute of Neurological Disorders and Stroke, Bethesda, USA

**Keywords:** Stroke, Rehabilitation, Stroke Recovery, Cerebral Ischemia, Intracerebral Hemorrhage, Modified Rankin Score

## Abstract

**Background:**

Many stroke recovery interventions are most beneficial when started 2-14d post-stroke, a time when patients become eligible for inpatient rehabilitation facilities (IRF) and neuroplasticity is often at its peak. Clinical trials focused on recovery need to expand the time from this plasticity to later outcome timepoints.

**Methods:**

The disability course of patients with acute ischemic stroke (AIS) and intracranial hemorrhage (ICH) enrolled in Field Administration of Stroke Therapy Magnesium (FAST-MAG) Trial with moderate-severe disability (modified Rankin Scale [mRS] 3–5) on post-stroke day4 who were discharged to IRF 2-14d post-stroke were analyzed.

**Results:**

Among 1422 patients, 446 (31.4%) were discharged to IRFs, including 23.6% within 2-14d and 7.8% beyond 14d. Patients with mRS 3–5 on day4 discharged to IRFs between 2-14d accounted for 21.7% (226/1041) of AIS patients and 28.9% (110/381) of ICH patients, (*p* < 0.001). Among these AIS patients, age was 69.8 (± 12.7), initial NIHSS median 8 (IQR 4–12), and day4 mRS = 3 in 16.4%, mRS = 4 in 50.0%, and mRS = 5 in 33.6%. Among these ICH patients, age was 62.4 (± 11.7), initial NIHSS median 9 (IQR 5–13), day 4 mRS = 3 in 9.4%, mRS = 4 in 45.3%, and mRS = 5 in 45.3% (*p* < 0.01 for AIS vs ICH). Between day4 to day90, mRS improved ≥ 1 levels in 72.6% of AIS patients vs 77.3% of ICH patients, *p* = 0.3. For AIS, mRS improved from mean 4.17 (± 0.7) to 2.84 (± 1.5); for ICH, mRS improved from mean 4.35 (± 0.7) to 2.75 (± 1.3). Patients discharged to IRF beyond day14 had less improvement on day90 mRS compared with patients discharged between 2-14d.

**Conclusions:**

In this acute stroke cohort, nearly 1 in 4 patients with moderate-severe disability on post-stroke day4 were transferred to IRF within 2-14d post-stroke. ICH patients had nominally greater mean improvement on mRS day90 than AIS patients. This course delineation provides a roadmap for future rehabilitation intervention studies.

## Background

Stroke is one of the leading causes of serious long-term disability in the United States. More than one-quarter of new stroke patients develop impairment in basic activities of daily living, and more than half have reduced mobility [1]. In recent years, reperfusion therapies for acute ischemic stroke (AIS) with intravenous thrombolysis and mechanical thrombectomy have reduced the rate of disability among stroke survivors. However, these treatments are only available to a small proportion of patients and up to three-quarters of patients so treated nevertheless have long-term disability [2].

Stroke rehabilitation has been the mainstay therapy to reduce disability after stroke, and it has been shown that high-intensity physical therapy enhances post-stroke recovery [3–5]. However, among stroke patients admitted to acute inpatient rehabilitation facility (IRF), long-term outcome remains suboptimal, with more than 30% of patients having low functional outcome scores indicating the need for continued assistance for mobilization or activities of daily living [6].

New trials are increasingly assessing innovative pharmacological and device therapies to stimulate neuroplasticity for patients with AIS and intracerebral hemorrhage (ICH) to further enhance neuro-recovery [7]. As the first week after ictus represents a time period of particularly substantial neuroplasticity [8], trials of diverse therapies are increasingly targeting recovery-enhancing treatment start towards the end of the acute admission and the very beginning of the acute rehabilitation stay. For example, recent trial of neural progenitor cells, pharmacologic agents, and neuro-modulatory therapies have sought to enroll patients in days 2–14 post-stroke timeframe [9, 10].

The design and conduct of these trials are challenged by a paucity of information regarding the course and outcome of end-of-acute stay stroke patients planned for transfer to an IRF for acute rehabilitation. Among outcome measures, more information is needed especially regarding the evolution of global disability on the modified Rankin Scale (mRS). The mRS is the standard lead outcome measure in acute stroke trials and desirable to include in trials evaluating patients enrolled toward the end of their acute stay. However, the mRS has been assessed only infrequently in stroke recovery trials. Therefore, it is of utmost importance to investigate the trajectory and final global disability of patients discharged to an IRF for physical, occupational and speech therapies after stroke. A population of particular interest are patients with moderate-severe (mRS 3–5) disability at the time at which early recovery interventions may be initiated in clinical trials.

In this study, we evaluated the trajectory of the patients who had moderate-severe disability, determined using their day 4 mRS, and who were discharged to IRF. We also investigated the differences in disability and its trajectory between patients with AIS and ICH.

## Methods

This was a secondary analysis of patients enrolled in the National Institute of Health Field Administration of Stroke Therapy Magnesium (FAST-MAG) Trial. FAST-MAG was a phase 3 placebo-controlled randomized clinical trial conducted from 2005 to 2013 at 60 stroke-receiving hospitals in two large California counties [11]. This study was performed in accordance with relevant guidelines and regulations approved by the institutional review boards of each hospital study site, including the IRB of the overall study coordinating center at UCLA (IRB# 13–000413). On-scene competent patients or legally authorized representatives provided explicit written informed consent; when the patient was not competent and no legally authorized representative was present, patients were enrolled, with the approval of each site's IRB, under United States regulations for waiver of informed consent for research performed in emergency circumstances.

### Patients

For the current study, we identified in the FAST-MAG database patients meeting the following criteria: 1) final diagnosis of AIS and ICH; 2) moderate-severe disability (modified Rankin Scale (mRS) global disability score 3–5) on day 4 post-stroke; 3) discharged to IRF between days 2–14. Since the primary trial results showed a neutral effect of magnesium sulfate on the outcome, without an interaction effect between the treatment group and mRS score, we combined the placebo and magnesium groups in the analysis.

### Clinical data

We extracted patient demographics characteristics (age, sex, race, ethnicity, pre-stroke mRS), past medical history (hypertension, diabetes mellitus, hyperlipidemia, any alcohol consumption, any tobacco use, previous history of stroke, cardiac disease-including coronary artery disease or myocardial infarction), presenting and day 4 deficit severity on the National Institutes of Health Stroke Scale (NIHSS), infarct size at presentation on the Alberta Stroke Program Early CT Score (ASPECTS) scale (for patients with AIS only) or volume in ICH patients, and time from hospital arrival to discharge to IRF in days.

The lead outcome was mRS score at 3 months. All final mRS assessments were performed by physician and nurse raters certified in the validated Rankin Focused Assessment method for assigning modified Rankin Scale scores. For the day 4 mRS, raters were encouraged but not required to use the Rankin Focused Assessment. In addition, rater instructions for the day 4 mRS were that the score is based on the rater’s judgment of what the subject could do on day 4, using not only patient report, but also available information from family, physicians and nurses, physical and occupational therapists, and the rater’s direct assessment including an NIHSS and a Barthel Index evaluation. Raters were directed that the scores should indicate their best judgment of what activities the patient could do on day 4, not simply what the patients had had an opportunity to do so far. At 3 months, the Glasgow Outcome Scale, Barthel Index of activities of daily living, and NIHSS were also assessed.

The modified Rankin Scale includes death in its spectrum of outcomes, as mRS level 6. Accordingly, patients who died between discharge from IRF and day 90 were scored as mRS 6 for all visits subsequent to their death.

### Statistics

Descriptive data are presented as mean ± standard deviation for normally distributed continuous variables, both median and interquartile range and mean ± standard deviation for ordinal variables and non-normally distributed continuous variables, and number (frequency) for nominal variables. The Student’s t-test, Mann–Whitney U-test, and Fisher exact test were used for comparison of data wherever appropriate. Two-sided significance tests were implemented throughout, and a threshold of *p* < *0.05* was considered statistically significant. As this is a secondary analysis of the trial dataset, all analyses were considered exploratory. 

## Results

**Fig. 1:**
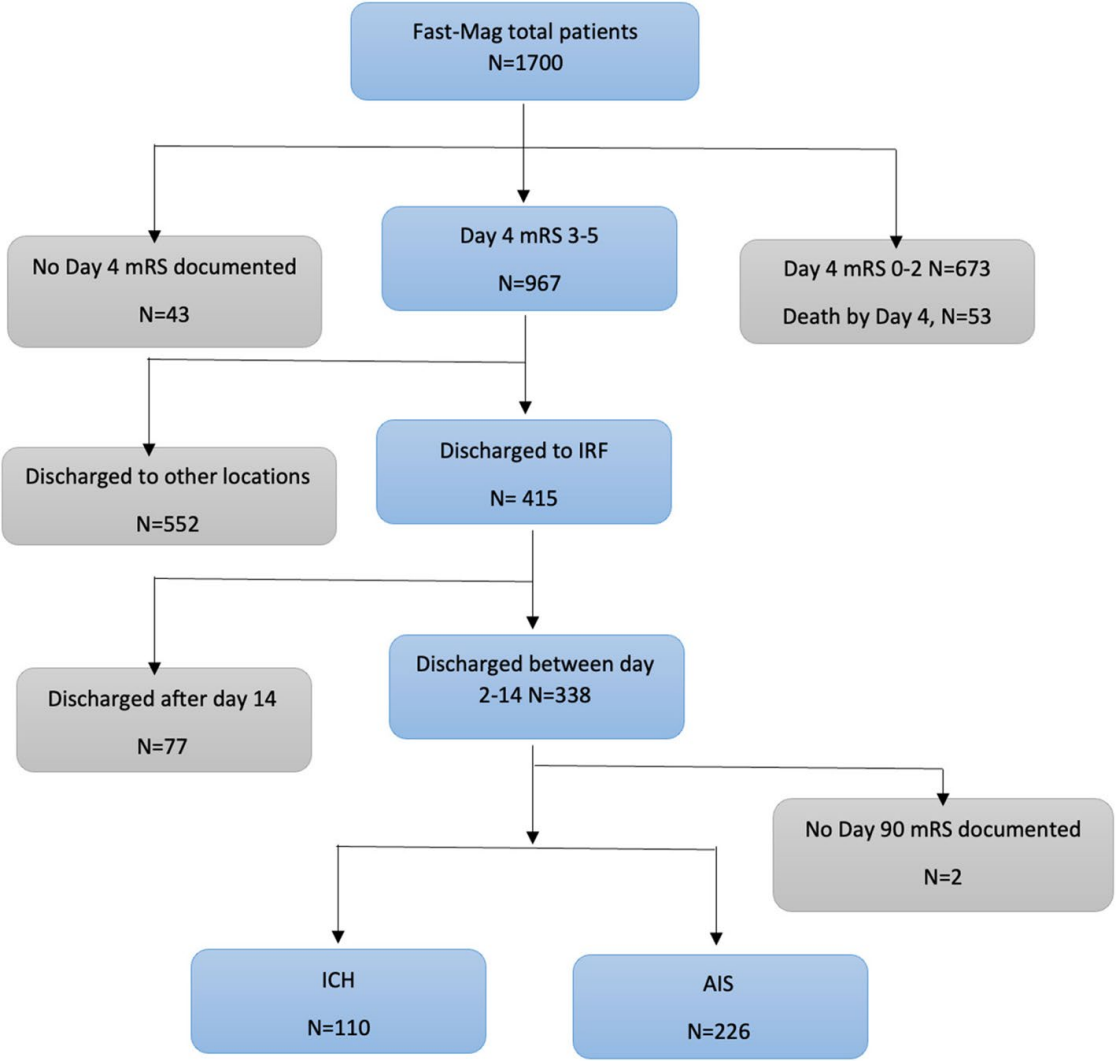
Flow chart of patient selection for the current study

Among 1700 patients enrolled in the FAST-MAG trial, 1041 patients had final diagnosis of acute ischemic stroke (AIS) and 381 patients had final diagnosis of intracranial hemorrhage (ICH). Patient selection for the current study is shown in the flow chart in Fig. 1. Among patients with AIS, 294/1041 (28.2%) were discharged to IRF, while 152/381 (39.9%) of patients with ICH were discharged to IRF (*P* < *0.001*). Among the patients with AIS discharged to IRF, 259/294 (88.1%) were discharged between day 2–14; among the patients with ICH discharged to IRF, 112/152 (73.7%) were discharged to IRF between day 2–14 (*P* < *0.001*). Patients moderately-severely disabled (mRS 3–5) on day 4 discharged to IRFs between days 2–14 accounted for 21.7% (226/1041) of patients with AIS and 28.9% (110/381) of patients with ICH, (*p* < *0.001)*.

The demographics and clinical characteristics of patients who were discharged to IRF between days 2–14 stratified by stroke type are shown in Table 1. Patients with AIS compared with patients with ICH were significantly older and less often Hispanic/Latino. Patients with AIS more often had cardiovascular risk factors of hyperlipidemia and coronary artery disease, though not hypertension or diabetes, and less often had history of alcohol use. In addition, patients with AIS had significantly lower admission and day 4 NIHSS scores, though slightly more frequently had pre-stroke mRS higher than 0. Patients with AIS had a shorter length of stay, a half-day shorter when comparing the two medians.

**Table 1:** Demographic and Clinical Characteristics of Patients with AIS and ICH with day 4 mRS 3-5 who were discharged to IRF between day 2-14

	AIS	ICH	P value
N=336	226	110	
Age, Mean (SD)	69.8 (12.7)	61.4 (11.8)	<.001
Sex, female, N (%)	94 (41.6)	36 (32.7)	0.11
Race			
White, N (%)	163 (72.1)	85 (77.3)	0.08
Black/African American, N (%)	40 (17.7)	10 (9.1)	
Asian, N (%)	22 (9.7)	14 (12.7)	
American Indian/Alaskan Native, N (%)	1 (0.4)	0	
Native Hawaiian/Pacific Islander, N (%)	0	1 (0.9)	
Hispanic Ethnicity, Hispanic, N (%)	42 (18.6)	42 (38.2)	<.001
HTN, N (%)	176 (77.9)	83 (75.5)	0.68
DM, N (%)	51 (22.6)	20 (18.2)	0.39
Heart disease (CAD+MI), N (%)	57 (25.2)	5 (4.5)	<.001
Hyperlipidemia, N (%)	110 (48.7)	31 (28.2)	<.001
Prior history of stroke or TIA, N (%)	33 (14.6)	9 (8.2)	0.11
Tobacco use, N (%)	41 (18.1)	23 (20.9)	0.55
Any alcohol use, N (%)	23 (24.5)	24 (40)	0.049
IV-tPA, N (%)	106 (46.9)		
Endovascular Therapy, N (%)	8 (3.5%)		
NIHSS at hospital arrival, Mean (SD)	11.5 (6.7)	13 (6.8)	0.06
Median (IQR)	11 (6-17)	12 (8-15)	0.10
NIHSS at day 4, Mean (SD)	8.35 (5.3)	9.95 (6.1)	0.01
Median (IQR)	8 (4-12)	9 (5-13)	0.03
Barthel Index day 4, Mean (SD)	33.3 (24.5)	28.3 (21.7)	
Median (IQR)	30 (15-50)	25 (10-45)	0.10
Glasgow Outcome Scale day 4, Mean (SD)	2.84 (0.37)	2.88 (0.35)	
Median (IQR)	3(3-3)	3 (3-3)	0.43
Modified Rankin Scale day 4, Mean (SD)	4.17 (0.69)	4.35 (0.66)	0.03
Median (IQR)	4 (4-5)	4 (4-5)	0.03
Initial ASPECTS score in AIS patients, volume in ICH patients			
Mean (SD)	8.06 (2.66)	18.4 (22.8)	--
Median (IQR)	10 (6-10)	9.6 (4.6-23)	
Pre-stroke mRS 0, N (%)	197 (87.2)	105 (95.4)	0.018
Length of stay, Mean (SD)	6.28 (2.85)	7.34 (2.94)	
Median (IQR)	6 (4-8)	6.5 (5-10)	0.002

**Fig. 2:**
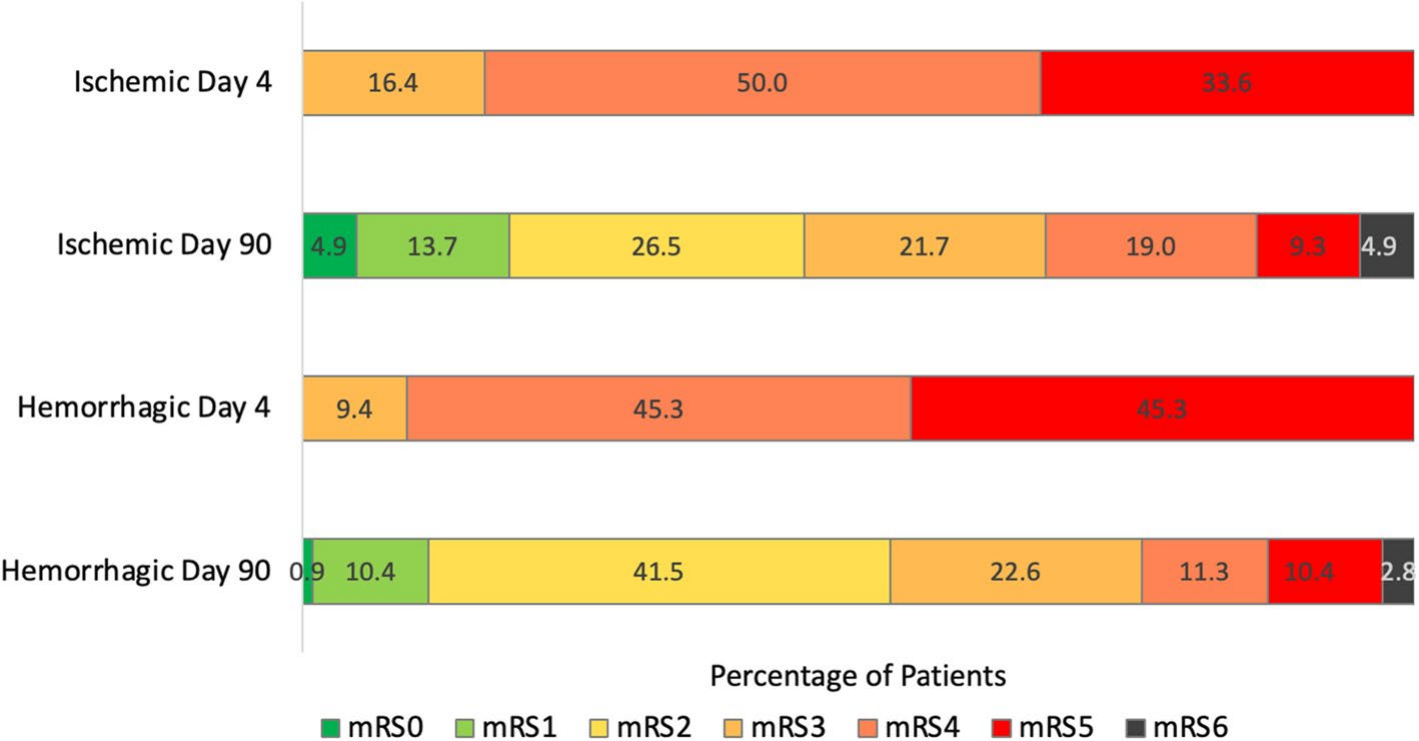
The evolution of global disability from day 4 to day 90 among patients with AIS and ICH with day 4 mRS 3–5 discharged to inpatient rehabilitation facilities between days 2–14

The level of disability among patients at day 4 who were discharged to IRF between days 2–14 is shown in Fig. 2. On day 4 post-stroke, 16.4% had mRS 3, 50.0% had mRS 4 and 33.6% had mRS 5. Among patients with ICH discharged to IRF between days 2–14, 9.4% had mRS 3, 45.3% had mRS 4 and 45.3% had mRS 5 on day 4 post-stroke. The trajectories of mRS outcomes between day 4 and day 90 in the populations with AIS and ICH are shown in Table 2 and Fig. 2. Between day 4 and day 90, mRS improved by 1 or more levels in 72.6% of AIS patients vs 77.3% of ICH patients, *p* = *0.3*. For patients with AIS, the mRS improved from mean 4.17 (± 0.7) to 2.84 (± 1.5) on day 90, and from median 4 (4–5) to 3 (2–4); for patients with ICH, the mRS improved from mean 4.35 (± 0.7) to 2.75 (± 1.3) on day 90, and from median 4 (4–5) to 2 (2–3). For both mean/median and dichotomized outcomes at day 90, tendencies to less favorable scores for AIS vs ICH did not reach statistical significance (Table 2).

**Table 2:** Day 90 Outcomes of the Patients with AIS and ICH with Day 4 mRS 3-5 who were discharged to IRF on day 2-14

	AIS	ICH	*P* value
mRS Change Day 4 to Day 90			
All patients, mean (SD)	-1.34 (1.37)	-1.60 (1.18)	0.08
Patients with mRS 3 on Day 4, mean (SD)	-1.19 (1.24)	-1.10 (0.88)	0.83
Patients with mRS 4 on Day 4, mean (SD)	-1.32 (1.44)	-1.65 (1.08)	0.16
Patients with mRS 5 on Day 4, mean (SD)	-1.43 (1.33)	-1.67 (1.31)	0.34
Day 90 mRS Outcomes			
Nondisabled, mRS 0–1, N (%)	42 (20.3)	12 (11.3)	0.09
Independent, mRS 0–2, N (%)	102 (45.1)	56 (52.8)	0.19
Ambulatory, mRS 0–3, N (%)	151 (66.8)	80 (75.5)	0.11
mRS score			
Mean (SD)	2.8 (1.5)	2.8 (1.3)	
Median (IQR)	3 (2–4)	2 (2–3)	0.53
Mortality, (mRS 6) N (%)	11 (4.8)	3 (2.8)	0.39
Barthel Index^a^			0.21
Day 90 Mean (SD)	75.9 (28.9)	80.5 (26.8)	
Median (IQR)	90 (60, 100)	90 (75, 100)	
Glasgow Outcome Scale^b^			0.08
Day 90 Mean (SD)	2.32 (1.02)	2.12 (0.90)	
Median (IQR)	2 (1,5)	2 (1,5)	

^a^Barthel Index day 90 scores were missing in 13/226 (5.8%) AIS patients and 9/110 (8.2%) ICH patients

^b^Glasgow Outcome Scale scores were missing in 1/226 (0.4%) and 5/110 (4.5%)

**Fig. 3:**
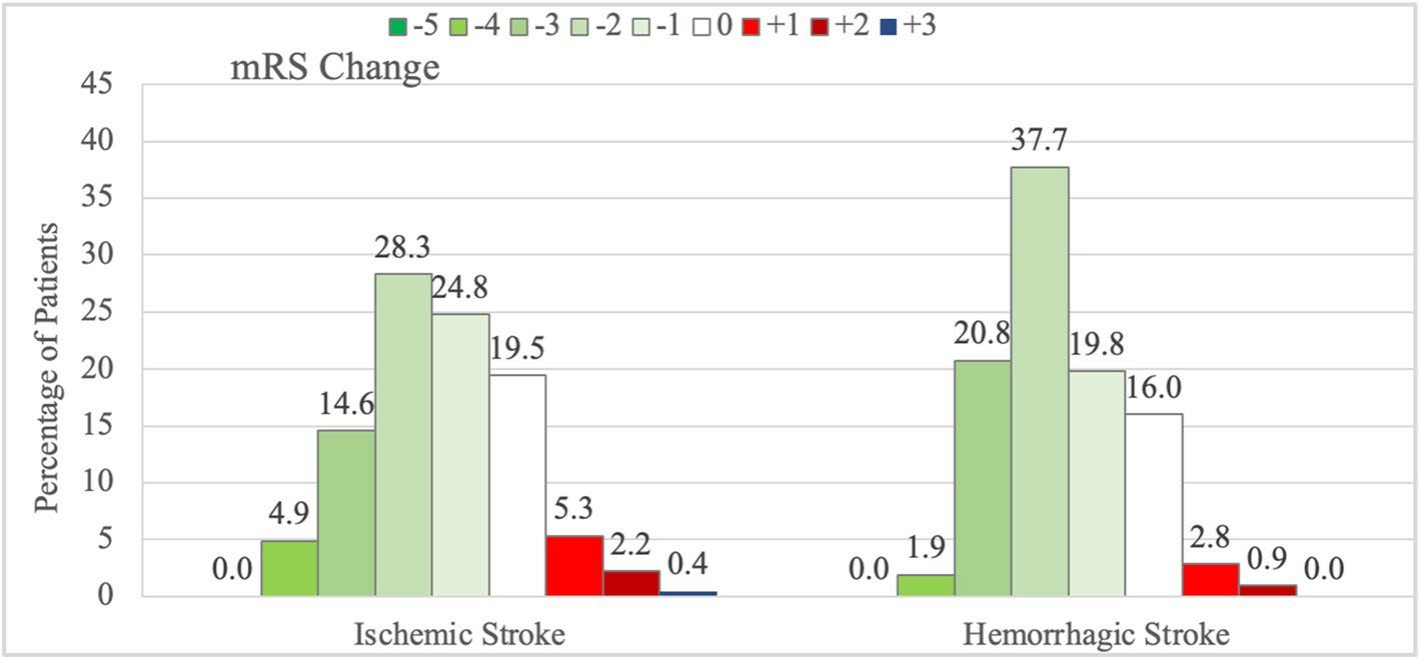
Modified Rankin Scale change values from day 4 to day 90 for patients with AIS and ICH. In both groups, more patients improved than worsened, with a greater proportion improving in the ICH group

However, considering that patients with AIS began from a better overall level, the magnitude of change in disability did differ between the groups. Patients with AIS had less improvement from day 4 to day 90 with patients with ICH, delta of 1.34 vs 1.60, *p* = *0.08.* As seen in Fig. 3, 72.6% of patients with AIS improved on the mRS from day 4 to day 90 compared with 80.2% of patients with ICH. For both, the most common amplitude of improvement was by 2 levels on the mRS, with a sharper peak for patients with ICH. In contrast, 7.9% of patients with AIS worsened on the mRS from day 4 to day 90 compared with 3.7% of patients with ICH.

**Fig. 4:**
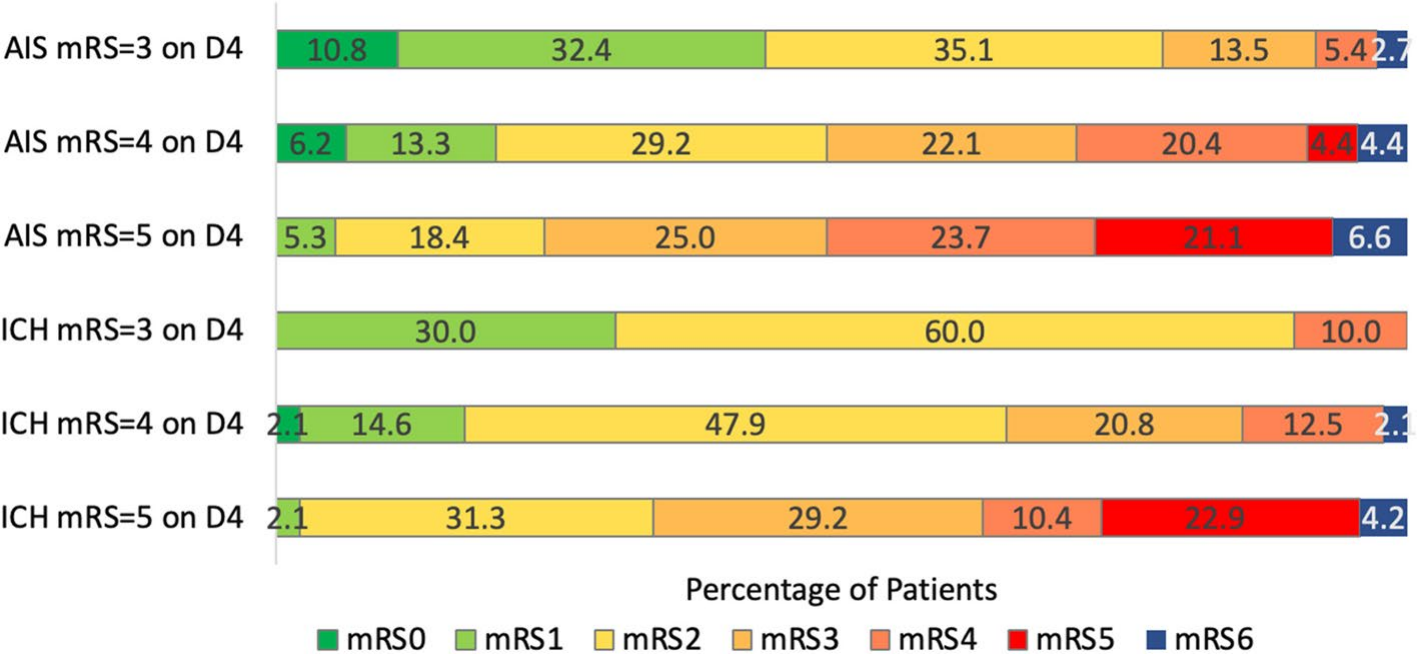
Day 90 global disability distributions among patients with AIS and ICH discharged to inpatient rehabilitation facilities between days 2–14, separately for patients with day 4 mRS 3, day 4 mRS 4, and day 4 mRS 5

Similarly, neurologic deficits on the NIHSS improved to a lesser degree among patients with AIS (from 8.4 at day 4 to 6.4 at day 90) than among patients with ICH (from 10.0 at day 4 to 4.8 at day 90), delta –1.83 (± 8.8) vs –5.09 (± 8.1), *p* = *0.002.* The day 90 mRS outcome distributions separately for patients with mRS 3 at day 4, mRS 4 and day 4, and mRS 5 at day 4 are shown in Fig. 4. Patients with mRS 4 at day 4 had the most distributed range of day 90 outcomes. Patients with mRS 3 at day 4 had higher frequencies of nondisabled mRS 0–1 day 90 outcomes, 43.2% for patients with AIS and 30.0% for patients with ICH. Patients with mRS 5 at day 4 had higher frequencies of severely disabled or dead (mRS 5–6) day 90 outcome, 27.7% for patients with AIS and 27.1% for patients with ICH.

There was no significant interaction of initial ASPECTS scores with mRS change from day 4 to day 90 among ischemic stroke patients, but analytic power was constrained by the high frequency of normal ASPECTS scores in the hyperacutely imaged cohort. There was also no significant interaction of initial ICH volume with mRS change from day 4 to day 90.

Among the smaller group of patients discharged to IRF beyond day 14, the day of discharge was median 20.5 days (IQR 17–31.5) for patients with AIS and 25 days (IQR 18–31) for patients with ICH. Patients who were discharged to IRF beyond day 14 had less improvement between day 4 and day 90 and worse day 90 mRS outcomes than did patients discharged to IRF between days 2–14. (Table 3).

**Table 3:** Recovery Trajectories with Differing IRF Discharge Time Groups in the Patients with AIS and ICH with mRS 3-5 on Post-stroke Day 4

	AIS Discharged to IRF on day 2–14 (*N* = 226)	AIS Discharged to IRF > Day 14 (*N* = 34)	P Value	ICH Discharged to IRF On day 2–14 (*N* = 110)	ICH Discharged to IRF > day 14 (*N* = 40)	*P* Value
mRS day 4 Mean (SD)	4.17 (0.7)	4.85 (0.4)	< .001	4.35 (0.7)	4.88 (0.3)	< .001
mRS day 4 Median (IQR)	4 (4–5)	5 (5–5)	< .001	4 (4–5)	5 (5–5)	< .001
mRS day 90 Mean (SD)	2.84 (1.5)	3.65 (1.3)	0.003	2.75 (1.3)	3.90 (1.1)	< .001
mRS day 90 Median (IQR)	3 (2,4)	3 (3,5)	0.002	2 (2,3)	4 (3,5)	< .001
Change mRS day 4 to day 90, Mean (SD)	-1.34 (1.4)	-1.21 (1.2)	0.60	-1.60 (1.2)	-0.97 (1.2)	0.35
Day 90 mRS 0 (%)	4.9	0	0.02	0.9	0	< .001
Day 90 mRS 1 (%)	13.7	5.9	10.4	0		
Day 90 mRS 2 (%)	26.5	11.8	41.5	20		
Day 90 mRS 3 (%)	21.7	26.5	22.6	7.5		
Day 90 mRS 4 (%)	19	26.5	11.3	35		
Day 90 mRS 5 (%)	9.3	26.5	10.4	37.5		
Day 90 mRS 6 (%)	4.9	2.9	2.8	0		

Among 226 patients with AIS who were discharged to IRF between days 2–14, site of residence on day 90 was home in 82.3%, acute rehabilitation in 2.7%, skilled nursing facility in 4.0%, acute care hospital in 2.2%, other in 8.9%, and 4.9% of patients had died. Among 110 patients with ICH who were discharged to IRF between days 2–14, site of residence on day 90 was home in 75.4%, acute rehabilitation in 6.4%, skilled nursing 7.3%, acute care hospital in 0.9%, other in 1.0%, and 2.7% of patients had died.

## Discussion

In this study, substantial proportions of hyperacute stroke patients presenting with motor deficits were discharged to an acute inpatient rehabilitation facility, including more than one-quarter of patients with AIS and about 4 of every 10 patients with ICH. Among patients bound for an IRF between days 2–14, with moderate to severe disability on day 4, when an early intervention targeting recovery might be initiated, the levels of day 4 disability were severe, with nearly 9 of every 10 patients having mRS levels of 4–5 and 1 of every 10 mRS of 3. Disability levels generally improved substantially between day 4 to day 90, with median improvement by about 1.5 mRS steps. Nonetheless, only a small minority of patients, 11–20%, achieved a nondisabled (mRS 0–1) outcome by day 90. Patients with ICH, compared with patients with AIS, had more severe deficits at day 4 but tended to improve to a greater degree through day 90. In addition, patients discharged to IRF beyond day 14 had less improvement and worse day 90 mRS outcomes compared to patients discharged to IRF between days 2–14.

The results of this study have direct implications for the design of clinical trials of neuro-recovery interventions begun in the subacute period, 2–14 days after stroke onset. This is an epoch that is being increasingly targeted for the start of a restorative intervention because it is a time period of particularly enhanced neuroplasticity [12, 13]. Recent trials of pharmacologic agent [14], progenitor cell [15], and neuromodulation therapies [16] have started interventions in this period. The design of subacute neuro-recovery trials will be aided by the data in this report as hitherto trial planning has been hampered by a paucity of information regarding patient course from this timepoint forward with conventional care among patients transferred or bound for transfer to inpatient rehabilitation facilities. The preponderance of IRF data on patient trajectory describes patient course from the baseline of admission or discharge from the inpatient facility through the next months or years [17]. These studies have often used highly detailed assessments, like the Functional Independence Measure, to characterize patient baseline [18–20]. Such extensive assessments cannot practicably be carried out in many patients early after stroke.

The design of early recovery studies has also been constrained by a lack of data regarding the evolution of the mRS global disability outcome in this patient population. The preponderance of recovery trials to date have enrolled patients in later subacute and chronic post-stroke periods. At those timepoints, the profile of a patient’s deficits has reached a somewhat stable plateau, permitting targeting of domain-specific functional impairments, such as hand function, leg function, aphasia, and hemispatial neglect. Highly focused domain-specific measures are then used to assess treatment effect [21]. However, in the 2–14 days period, patient deficits are often not yet stabilized. Over the subsequent course, a wide range of deficits might evolve to be the leading causes of functional limitation in a manner not fully predictable. In this setting, a global disability outcome measure like the mRS, sensitive to multiple domains of impairment has advantages [22]. This broad scope is one of the reasons that the mRS is the most common endpoint in trials of interventions begun in the acute period, 3–24 h after onset. For the same reason, the mRS may be a useful outcome measure for early-initiated recovery therapies, complementing the details provided by domain specific endpoints [21, 23]. The mRS also brings the added advantage of widely recognized clinical significance for patients, families, regulatory bodies, and payers.

The time course of stroke recovery varies tremendously across patients. Recovery is a complex process involving restoration of function in damaged neural tissue, reorganizing neural pathways, and relearning lost or impaired function [3]. Language recovery may continue for months beyond the time when upper extremity motor recovery has reached a plateau [12]. Given this nonlinear process of stroke recovery, a range of timepoints 3–12 months post-stroke are potentially useful to assess patient outcome after a recovery intervention. The current study evaluated patients through 3 months, an option for which extensive supporting information is available from acute treatment trials that standardly use this timepoint. Long-term cohort studies have shown that about 70% of a patient's maximum possible improvement is attained at 3 months following stroke [22]. The current study’s findings of a tendency to greater functional recovery for patients with ICH compared with AIS is consonant with several prior studies [24–27]. Differences in brain injury processes likely contribute to the course differences. In AIS, all tissue in the cerebral infarct is permanently injured. In ICH, dysfunction is in part due to compression of surrounding tissue by the hematoma mass and surrounding edema. As the perihematomal tissue is not destroyed, it can return to functionality as the blood is resorbed. In addition, patients with ICH on average are younger and have less cardiovascular risk factors than patients with AIS. They therefore may have more intrinsic neuroplasticity as well potentially an ability to participate more fully in therapy modalities [25, 28].

This study has limitations. First, the study baseline mRS assessment for all patients was on day 4 post-stroke. The trajectory of mRS scores may differ for patients enrolled in early recovery studies before or after this time-point. Second, patients were cared for in many different IRFs, and so rehabilitation therapies may have differed somewhat across sites. However, such variation suggests that current results reflect the broad range of conventional care encountered in routine clinical practice and so likely generalize well. Third, patients with pre-existing disability before index stroke were excluded from the population cohort. The evolution of their global disability during the first 3 post-stroke months in such patients may be somewhat worse than patients with pre-existing disability. Fourth, all study patients were enrolled in a clinical trial and so may not be fully representative of completely unselected patients. However, the FAST-MAG trial had very broad entry criteria. In addition, for the purpose of planning future clinical trials, analyzing patients who have been enrolled in an actual clinical trial is advantageous. Lastly, this study analyzed the mRS, which is a broad, ordinal scale, rather than more fine-grained outcome measures like the Functional Independence measure (FIM). Advantages of the mRS include that all step changes are clinically meaningful and mRS outcomes are well-documented in acute stroke trials. Analyzing FIM scores in a similar manner is desirable, but FIM scores were not collected in the FAST-MAG trial database.

## Conclusions

In conclusion, in a broad cohort of acute ischemic and hemorrhagic stroke patients, nearly one-quarter of patients were transferred to IRF within 2–14 days post-stroke. On average, between days 4 to 90, patients with AIS improved by 1.34 levels and patients with ICH by 1.60 levels on the mRS. Patients with ICH discharged to the IRF achieved a better clinical recovery despite worse presentation on admission. This delineation of the trajectory of mRS recovery under conventional rehabilitation care provides a roadmap for future rehabilitation intervention studies.

## Data Availability

The database of the main FAST-MAG trial is publicly available through the NIH-NINDS repository of Archived Clinical Research Datasets; url: https://www.ninds.nih.gov/current-research/research-funded-ninds/clinicalresearch/archived-clinical-research-datasets.

## Abbreviations

AIS: Acute Ischemic Stroke
ICH: Intracranial Hemorrhage
FAST-MAG: Field Administration of Stroke Therapy Magnesium
mRS: Modified Rankin Scale
IRF: Inpatient Rehabilitation Facility
NIHSS: National Institutes of Health Stroke Scale
ASPECTS: Alberta Stroke Program Early CT Score
